# Structure–Barrier–Release Relationships of Low-Temperature ALD ZrO_2_ Thin Films on Co–Cr Alloys: Reducing Long-Term Co and Cr Ion Release in Artificial Saliva

**DOI:** 10.3390/ma19194173

**Published:** 2026-09-29

**Authors:** Anna Ziębowicz, Wiktoria Groelich, Anita Kajzer, Wojciech Kajzer, Marta Kiel-Jamrozik, Julia Kolasa, Julia Lisoń-Kubica, Katarzyna Nowińska, Ada Orłowska, Zbigniew Paszenda, Gabriela Wielgus, Agnieszka Antończyk, Witold Walke, Bogusław Ziębowicz

**Affiliations:** 1 Department of Biomaterials and Medical Devices Engineering, Silesian University of Technology, 41-800 Zabrze, Polandanita.kajzer@polsl.pl (A.K.); wojciech.kajzer@polsl.pl (W.K.); marta.kiel-jamrozik@polsl.pl (M.K.-J.); julia.kolasa@polsl.pl (J.K.); julia.lison-kubica@polsl.pl (J.L.-K.); ada.orlowska@polsl.pl (A.O.); zbigniew.paszenda@polsl.pl (Z.P.); gabriela.wielgus@polsl.pl (G.W.); witold.walke@polsl.pl (W.W.); 2 Department of Electrical Engineering and Industrial Automation, Silesian University of Technology, 44-100 Gliwice, Poland; katarzyna.nowinska@polsl.pl; 3 Department of Materials Science and Artificial Intelligence in Dentistry Science, Medical University of Silesia in Katowice, 41-808 Zabrze, Poland; agnieszka.antonczyk@sum.edu.pl; 4 Department of Engineering Materials and Biomaterials, Silesian University of Technology, 44-100 Gliwice, Poland; boguslaw.ziebowicz@polsl.pl

**Keywords:** atomic layer deposition, zirconia, Co–Cr alloy, barrier coating, electrochemical impedance spectroscopy, cumulative ion release, surface engineering, biomaterials

## Abstract

**Highlights:**

- ALD ZrO_2_ reduced cumulative 28-day Co and Cr release from Co–Cr alloys in artificial saliva.
- Co and Cr release decreased across CAST, MILLED and PRINTED substrates after ZrO_2_ deposition.
- PRINTED + ZrO_2_ exhibited the lowest residual Co and Cr release, with Mo below the detection limit.
- EIS revealed manufacturing-dependent changes in barrier response after 28-day exposure.
- Cumulative ion release (µg/cm2) enables functional ranking of coating/substrate systems.
- 28-day cumulative ion release provides a practical functional descriptor of ALD barrier performance.
- Barrier effectiveness depends on the complete coating/interface/substrate system.
- Manufacturing route modulates the electrochemical response and residual metal-ion release.
- Low-temperature ALD ZrO_2_ restricts sustained Co and Cr transfer under simulated oral conditions.
- The structure–barrier–release framework supports the rational assessment of ceramic barriers for Co–Cr biomaterials.

**Abstract:**

Co–Cr alloys are widely used in biomedical devices owing to their excellent mechanical properties and corrosion resistance; however, sustained metal-ion release in physiological environments remains an important materials concern. Low-temperature atomic layer deposition (ALD) enables the formation of conformal ceramic barriers that can restrict long-term metal-ion transport. In this study, nominally ~50 nm ALD-derived ZrO_2_ coatings were deposited on Co–Cr substrates manufactured by investment casting (CAST), CAD/CAM milling (MILLED), and laser powder bed fusion (PRINTED). The coatings were characterized using scanning electron microscopy (SEM), atomic force microscopy (AFM), Raman spectroscopy, transmission electron microscopy with selected-area electron diffraction (TEM/SAED), and X-ray photoelectron spectroscopy (XPS), while the electrochemical response was evaluated by open-circuit potential (OCP) and electrochemical impedance spectroscopy (EIS). Long-term barrier performance was quantified by inductively coupled plasma atomic emission spectroscopy (ICP-AES) as cumulative Co and Cr release for all manufacturing routes and Mo release for the Mo-containing CAST and PRINTED alloys after 28 days in artificial saliva at 37 °C. ZrO_2_ deposition reduced cumulative Co and Cr release for all manufacturing routes. Mo release was also reduced in the Mo-containing alloys, with Mo remaining below the analytical detection limit (<0.005 µg cm^−2^) for the PRINTED + ZrO_2_ system. Electrochemical measurements further demonstrated manufacturing-route-dependent differences in barrier response between the initial and 28-day exposure states. Overall, the findings establish a structure–barrier–release framework linking coating formation and electrochemical response with cumulative ion release, and identify 28-day cumulative ion release as a practical functional descriptor for comparing the long-term performance of ALD-derived ceramic barriers on Co–Cr alloys.

## 1. Introduction

The long-term performance of metallic biomaterials is governed not only by their bulk mechanical properties but also by the stability of their surface under physiological exposure [1,2,3,4,5,6]. Among metallic systems used in prosthetic and biomedical applications, Co–Cr alloys are widely recognized for their excellent combination of high mechanical strength, wear resistance and corrosion resistance [2,3,4]. Nevertheless, prolonged exposure to aqueous electrolytes containing aggressive ions and complexing species may locally destabilize the passive surface film, resulting in sustained metal-ion transfer into the surrounding environment [5,6,7,8,9,10]. From a materials perspective, the long-term release of Co, Cr and Mo reflects the stability of the alloy/oxide interface, the integrity of the passive layer and the availability of transport pathways across the surface [5,6,7,8,9,10].

Ion release is therefore a functional descriptor of surface stability under service-like conditions [5,7,8,9,10]. It integrates several coupled processes—including electrochemical reactions, oxide growth and reconstruction, adsorption of ionic species and diffusion through defects or porous surface layers—into a single experimentally measurable outcome [5,7,8]. Importantly, even in the absence of visible corrosion damage, cumulative ion release during prolonged exposure may reveal subtle differences in barrier performance between material states and surface modifications, making it a practical metric for comparing manufacturing routes and surface-engineering strategies [8,9,10].

Among the available surface-engineering strategies, conformal ceramic barriers provide a promising approach for limiting long-term ion transport by physically separating the metallic substrate from the surrounding environment [11,12,13,14,15,16]. Low-temperature atomic layer deposition (ALD) is particularly attractive owing to its ability to produce highly conformal, thickness-controlled films with excellent step coverage, including on geometrically complex substrates [11,12,13,14]. ALD-derived ZrO_2_ combines these advantages with the chemical stability characteristic of zirconia-based ceramic systems [15,16,17,18,19].

Beyond the materials-related perspective, minimizing metal-ion release has become increasingly important from a regulatory standpoint. The European Medical Device Regulation (MDR 2017/745) places particular emphasis on biological safety, risk management and long-term clinical performance [20,21,22]. Consequently, surface-engineering strategies capable of limiting metallic ion transfer may contribute to the overall biological safety profile of metallic biomaterials intended for long-term use [1,5,16,21,22].

Although numerous studies have demonstrated improved corrosion resistance following surface modification of Co–Cr alloys [7,8,15,16,19], the relationship between barrier structure, interfacial integrity and long-term ion transport remains insufficiently understood. Most studies rely primarily on structural or short-term electrochemical descriptors, whereas cumulative metal-ion release is less frequently adopted as the principal functional endpoint [7,8,9,10,15,16,19]. Moreover, coating morphology, structural state, interfacial chemistry and long-term ion release are rarely integrated within a single mechanistic framework. The current state of knowledge and the scientific contribution of the present study are summarized in Table 1. Our previous investigations demonstrated the feasibility of low-temperature ALD-derived ZrO_2_ modification of prosthetic Co–Cr alloys and its influence on physicochemical, biological and protective surface characteristics [15,16,19].

This study establishes a structure–barrier–release framework for understanding the long-term performance of low-temperature ALD-derived ZrO_2_ ceramic barriers deposited on Co–Cr substrates manufactured by casting, milling and additive manufacturing. Structural, chemical and electrochemical evidence is integrated with cumulative Co, Cr and Mo release after 28 days of exposure to artificial saliva, with cumulative ion release adopted as the principal functional descriptor of long-term barrier performance.

## 2. Materials and Methods

### 2.1. Preparation of Functional Barrier Systems

The experimental design was conceived to evaluate whether ultrathin zirconium dioxide (ZrO_2_) coatings deposited by low-temperature atomic layer deposition (ALD) can function as effective diffusion barriers limiting the long-term release of metallic ions from cobalt–chromium (Co–Cr) dental alloys under simulated oral conditions. Rather than focusing solely on the coating itself, the study considered the complete coating/substrate system, assuming that barrier performance is governed not only by the properties of the deposited ceramic layer but also by the manufacturing route and microstructural characteristics of the underlying alloy.

Three commercially available Co–Cr dental alloys representing different manufacturing technologies were selected as substrate materials: Gialloy PA for conventional investment casting (CAST), CopraBond K for subtractive CAD/CAM milling (MILLED), and EOS CoCr RPD for additive manufacturing by direct metal laser sintering (PRINTED). The manufacturing routes were intentionally selected to provide substrates with distinct microstructural characteristics, enabling evaluation of the influence of substrate architecture on ZrO_2_ barrier formation and subsequent functional performance. The nominal chemical compositions of the alloys, according to the manufacturers, are summarized in Table 2. Although Co and Cr were the principal constituents of all three alloys, their minor alloying elements differed. In particular, the MILLED alloy contained W (8.5 wt.%) and no Mo in its nominal composition, whereas the CAST and PRINTED alloys contained Mo (5.5 and 5.1 wt.%, respectively).

Disc-shaped specimens of identical dimensions were prepared from each material to eliminate geometric effects during coating deposition, immersion, and subsequent analytical measurements. Prior to ALD processing, all specimens underwent an identical surface preparation protocol consisting of sequential grinding, polishing, and cleaning to obtain reproducible surface conditions and minimize the influence of initial surface variability tuoand contaminants on coating nucleation and growth.

Nominally ~50 nm ZrO_2_ coatings were deposited using a low-temperature thermal ALD process. Detailed information regarding precursor chemistry, deposition conditions, and process optimization has been reported in our previous studies [15,19] and is therefore not repeated here. The selected process enables the formation of conformal ceramic coatings with precise thickness control, consistent with the characteristic advantages of ALD [11,12,13,14]. In the present study, the deposited ZrO_2_ layer was regarded as a functional ceramic barrier designed to limit the transport of Co, Cr, and Mo ions from the metallic substrate into the surrounding environment during prolonged exposure to artificial saliva.

**Figure 1:**
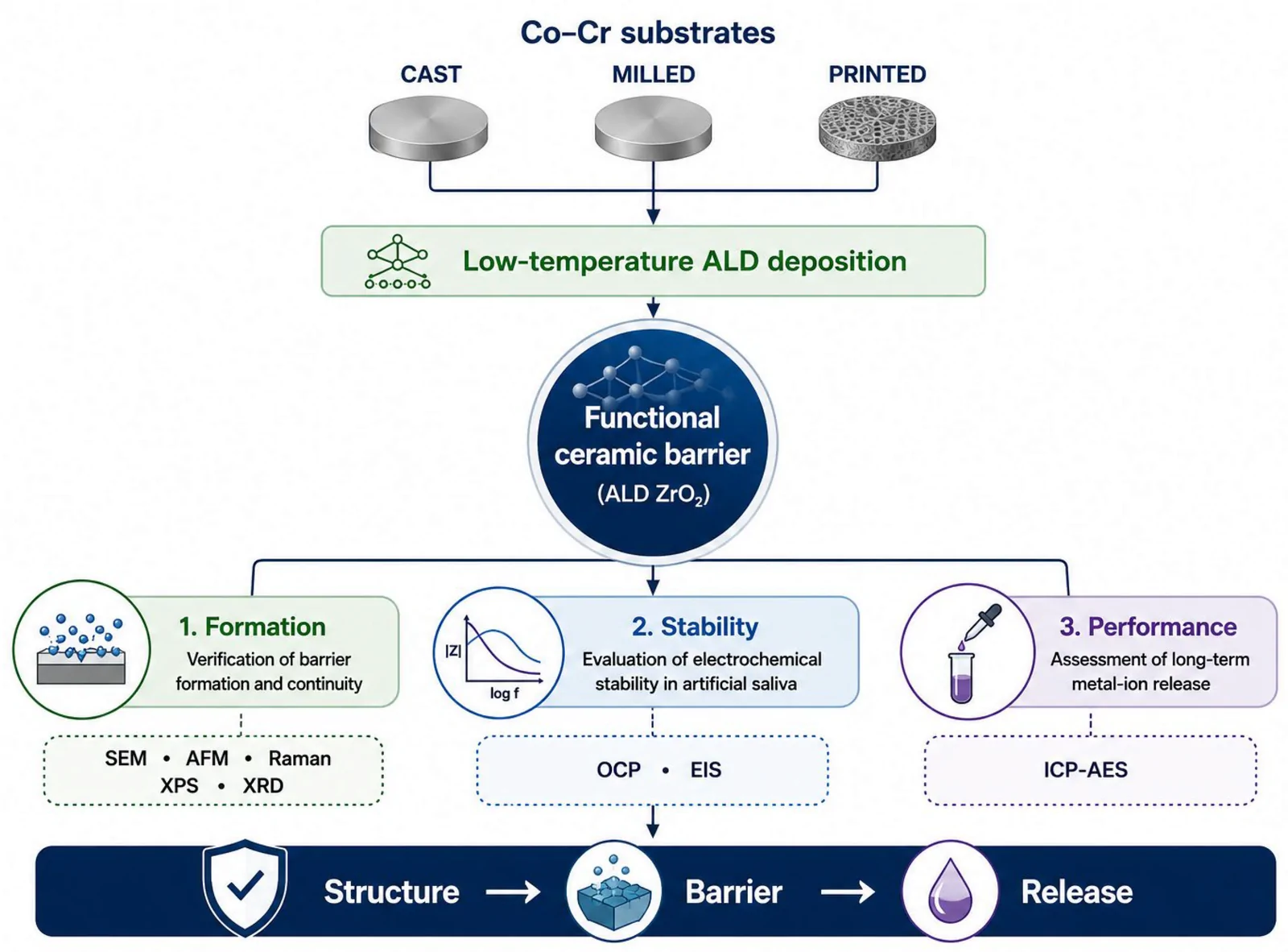
Experimental workflow for evaluating the formation, stability, and functional performance of nominally ~50 nm ALD-derived ZrO_2_ ceramic barriers deposited on Co–Cr alloys manufactured by investment casting (CAST), CAD/CAM milling (MILLED), and laser powder bed fusion (PRINTED). The integrated characterization strategy combines structural, chemical, electrochemical, and analytical methods within the proposed structure–barrier–release framework.

Following deposition, the specimens were subjected to the sequential characterization and immersion protocols described in the subsequent sections. The methodology was designed to verify barrier formation and structural integrity, assess electrochemical stability, and ultimately determine functional barrier performance through cumulative metal-ion release after 28 days of immersion in artificial saliva. The overall experimental workflow is presented in Figure 1 and forms the experimental basis of the proposed structure–barrier–release framework.

### 2.2. Verification of Barrier Formation and Structural Integrity

Following deposition, complementary microscopic, spectroscopic and surface-analytical techniques were employed to verify the formation, continuity and physicochemical characteristics of the nominally ~50 nm ALD-derived ZrO_2_ barrier. Because the integrity of an ultrathin ceramic coating cannot be established using a single analytical method, the characterization strategy combined morphological, topographical, structural and chemical evidence to provide a multidimensional assessment of barrier formation.

Surface morphology was examined by scanning electron microscopy (SEM) using a SUPRA 35 microscope (Carl Zeiss AG, Oberkochen, Germany) equipped with an energy-dispersive X-ray spectroscopy (EDS) system. Particular attention was paid to morphological homogeneity and the presence of localized surface defects or discontinuities that could potentially provide preferential pathways for electrolyte penetration.

Nanoscale surface topography was characterized by atomic force microscopy (AFM) using an XE-100 microscope (Park Systems, Suwon, Republic of Korea). Measurements were performed in resonance mode at 302 kHz using a silicon probe, and the recorded AFM images were analysed using XEI software (version 1.8.0, Park Systems, Suwon, Republic of Korea). AFM analysis was used to evaluate the nanoscale surface architecture of the coated specimens and to assess how the morphology associated with the different manufacturing routes was reflected in the surface topography following ALD.

The structural state of the deposited zirconia was evaluated by Raman spectroscopy. Spectra were analysed over the 110–750 cm^−1^ range and fitted using Gaussian–Lorentzian functions to distinguish the relative contributions of monoclinic (m), tetragonal (t) and cubic (c) ZrO_2_.

Cross-sectional characterization of the coating/substrate system was performed by transmission electron microscopy (TEM) and scanning transmission electron microscopy (STEM). Electron-transparent lamellae were prepared by focused ion beam (FIB) milling using a Helios NanoLab™ 600i SEM/Ga-FIB system (Thermo Fisher Scientific, formerly FEI, Hillsboro, OR, USA). TEM/STEM observations were performed using an S/TEM TITAN 80-300 microscope (Thermo Fisher Scientific, formerly FEI, Hillsboro, OR, USA) equipped with BF, DF and HAADF detectors. Cross-sectional observations were used to assess the continuity and nanoscale architecture of the deposited ZrO_2_ layer, while selected-area electron diffraction (SAED) was employed to verify its crystalline character.

The surface chemical composition and chemical state of the deposited barrier were investigated by X-ray photoelectron spectroscopy (XPS). Measurements included the O 1s, Zr 3d and C 1s core levels and the valence-band region. The ZrO_2_ surface was analysed both as received and after Ar^+^ ion cleaning performed under ultra-high-vacuum conditions at an ion energy of 0.7 keV for 1 min. Measurements were performed at an incidence angle of 45°, and surface charging was compensated using a floating electron gun. Atomic concentrations and spectral fitting were evaluated using MULTIPAK v9.9 (Physical Electronics, Inc., Chanhassen, MN, USA) and SIMPEAK 1.0 software.

Collectively, SEM, AFM, Raman spectroscopy, TEM/STEM–SAED and XPS provided complementary morphological, topographical, structural and chemical evidence of barrier formation. This characterization constituted the first experimental level of the workflow presented in Figure 1 and established the structural basis for the subsequent evaluation of electrochemical stability and long-term functional barrier performance.

### 2.3. Evaluation of Electrochemical Barrier Stability

Formation of a continuous ceramic barrier alone does not ensure long-term protective performance. Therefore, the second stage of the experimental workflow was designed to evaluate the electrochemical response of the ALD-derived ZrO_2_ barrier/substrate systems and to determine the effect of prolonged exposure to simulated oral conditions on their electrochemical characteristics.

Specimens were individually exposed to artificial saliva at 37 ± 1 °C for 28 days under identical experimental conditions. Electrochemical impedance spectroscopy (EIS) was performed for the investigated systems in the initial state and after the 28-day exposure period, enabling assessment of exposure-induced changes in the electrochemical response of the coating/substrate interface.

Electrochemical measurements were performed using a VoltaLab PGP 201 potentiostat (Radiometer Analytical SAS, Villeurbanne, France) equipped with a three-electrode electrochemical cell and operated using VoltaMaster 4 software (version 7.0.8, Radiometer Analytical SAS, Villeurbanne, France). The open-circuit potential (OCP) was determined after completion of the 28-day exposure period and was used to characterize the electrochemical state established at the barrier/electrolyte interface.

EIS measurements were performed at the open-circuit potential. The resulting impedance spectra were represented as Nyquist and Bode plots and analysed using equivalent electrical circuits selected according to the electrochemical response of the individual systems. The fitted circuit elements were used to characterize the resistance of the electrolyte (Rs), charge-transfer resistance (Rct), and, where applicable, resistance associated with electrolyte penetration through porous regions (Rpore). Constant-phase elements were introduced to describe non-ideal capacitive behaviour of the interfacial and porous layers.

Depending on the electrochemical response, equivalent-circuit models containing one or two time constants were applied. For systems exhibiting more complex interfacial behaviour, additional elements describing adsorption-related processes were included. The selection of different equivalent-circuit architectures reflected differences in the electrochemical characteristics of the investigated coating/substrate systems rather than the application of a single predefined model to all experimental groups.

Because the investigated systems exhibited different electrochemical responses and required different equivalent-circuit architectures, EIS parameters were interpreted mechanistically rather than subjected to direct statistical comparison. Changes in the impedance response between the initial state and the 28-day exposure condition were used to assess the influence of prolonged artificial-saliva exposure on the electrochemical behaviour of the barrier/substrate systems.

The electrochemical evaluation constituted the second experimental level of the workflow presented in Figure 1 and provided the intermediate link between structural verification of barrier formation and the subsequent functional assessment based on cumulative metal-ion release.

### 2.4. Functional Assessment of Barrier Performance

The functional performance of the ALD-derived ZrO_2_ barrier was evaluated by quantitative determination of metal-ion release during prolonged exposure to artificial saliva. Unlike structural and electrochemical characterization, which provide information on individual aspects of barrier behaviour, cumulative ion release was used as the principal functional endpoint reflecting the ability of the complete coating/substrate system to limit long-term metallic ion transport.

Uncoated and ZrO_2_-coated specimens were individually exposed to 0.1 dm^3^ of artificial saliva for 28 days under thermostatically controlled conditions at 37 ± 1 °C and pH 6.7 ± 0.2. After completion of the exposure period, the immersion solutions were collected and analysed for cobalt (Co), chromium (Cr), molybdenum (Mo), and zirconium (Zr).

Metal-ion concentrations were determined by inductively coupled plasma atomic emission spectroscopy (ICP-AES) using a JY2000 spectrometer (Horiba Jobin Yvon, Longjumeau, France). Calibration curves were established using certified reference materials supplied by Merck (Merck KGaA, Darmstadt, Germany).

The amount of released metal was normalized to the exposed specimen surface area and expressed as surface mass density (µg/cm^2^). This approach enabled direct comparison of ion release among the CAST, MILLED and PRINTED systems and between the corresponding uncoated and ZrO_2_-coated specimens.

Cumulative release of Co and Cr was determined for all investigated alloys, whereas Mo release was evaluated only for the Mo-containing CAST and PRINTED alloys. Mo was not applicable to the MILLED alloy because Mo is absent from its nominal composition (Table 2). Zr release was additionally monitored to assess the presence of zirconium originating from the deposited ceramic layer in the immersion medium. The quantitative ion-release results were subsequently interpreted together with the structural and electrochemical evidence to establish the relationship between barrier formation, electrochemical response and long-term functional performance.

Functional assessment by ICP-AES constituted the final experimental level of the workflow presented in Figure 1 and provided the direct quantitative endpoint of the proposed structure–barrier–release framework.

### 2.5. Data Analysis and Integrated Evaluation of Barrier Performance

The experimental strategy was designed to integrate complementary evidence obtained at the structural, electrochemical and functional levels. Each analytical method addressed a specific aspect of barrier performance: microscopic, spectroscopic and surface-analytical techniques were used to verify the formation and physicochemical characteristics of the ALD-derived ZrO_2_ layer; electrochemical measurements characterized the response of the barrier/substrate system before and after prolonged exposure to artificial saliva; and cumulative metal-ion release provided the principal functional endpoint of long-term barrier performance. The datasets were therefore interpreted collectively to establish relationships between barrier structure, electrochemical response and functional suppression of ion transport.

SEM, Raman spectroscopy, TEM/STEM–SAED and XPS data were used primarily to verify barrier morphology, structural state, crystallinity and surface chemistry, respectively, and were interpreted descriptively. AFM provided complementary nanoscale topographical information, including quantitative surface parameters where applicable.

Electrochemical impedance spectroscopy (EIS) data were analysed using equivalent-circuit models selected according to the electrochemical response of each investigated system. Changes between the initial and 28-day exposure conditions were interpreted on the basis of the fitted electrochemical parameters and corresponding circuit architectures. Because different equivalent-circuit models were required for the investigated systems, EIS parameters were interpreted mechanistically rather than subjected to direct statistical comparison. Open-circuit potential (OCP) values obtained after 28 days were used as an additional descriptor of the electrochemical state of the barrier/substrate systems.

Cumulative metal-ion release after 28 days was evaluated for Co, Cr, Mo and Zr and expressed as surface mass density (µg/cm^2^). Co and Cr release were adopted as the principal functional descriptors of barrier effectiveness, whereas Mo release provided complementary information on the transport of an additional alloying constituent. Zr release was considered separately as an indicator related to the behaviour of the deposited ZrO_2_ layer. Because the available ion-release data did not provide an appropriate basis for inferential statistical testing, the results were interpreted descriptively and comparatively among the investigated manufacturing routes and barrier conditions.

The integrated interpretation of structural, electrochemical and ion-release data provided the methodological basis for the proposed structure–barrier–release framework, in which barrier formation constitutes the structural level of evidence, electrochemical response represents the intermediate barrier-performance level, and cumulative metal-ion release provides the final functional endpoint.

### 2.6. The Use of AI in Images

**Figure 5:**
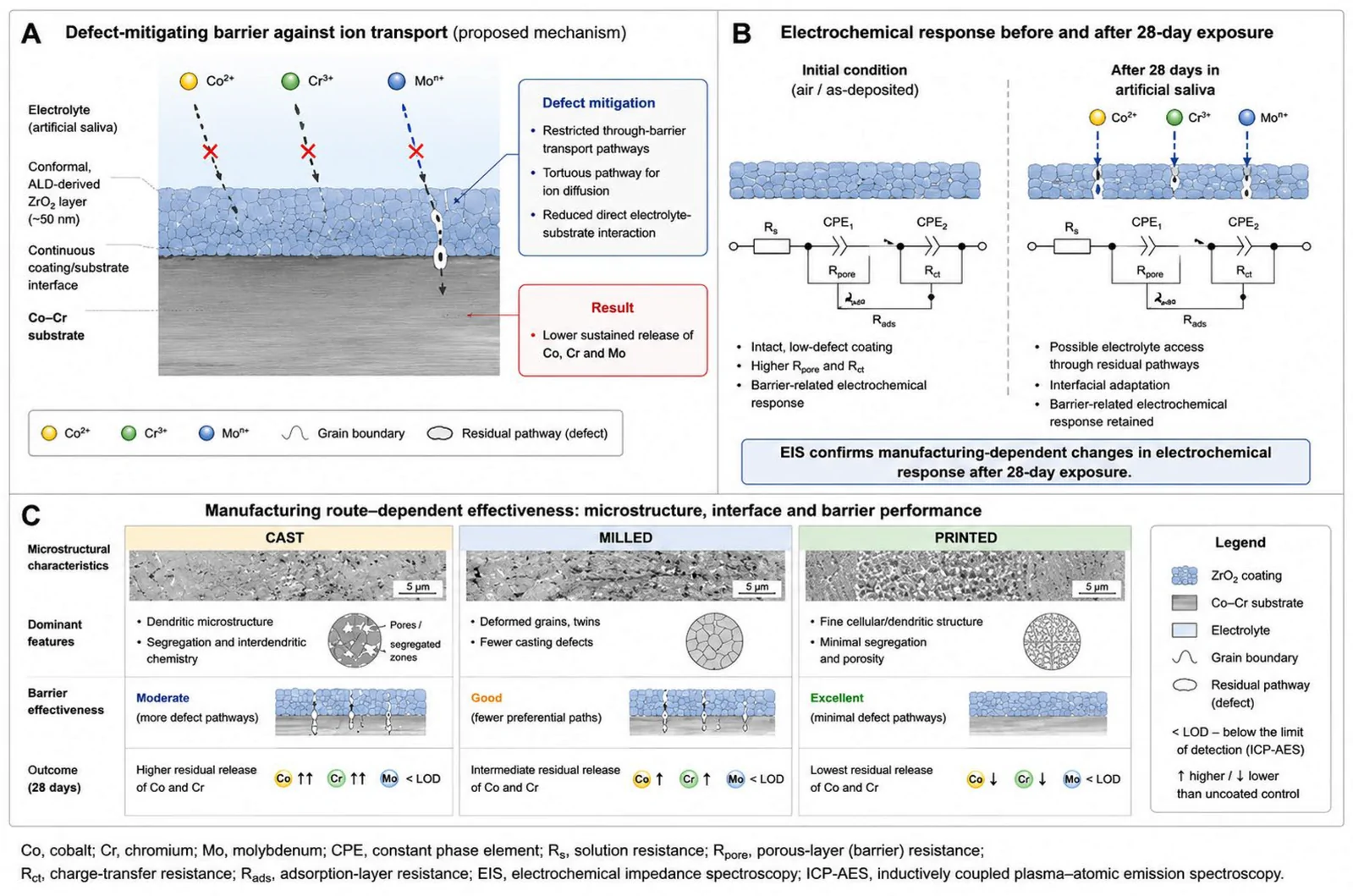
Schematic representation of the proposed structure–barrier–release mechanism governing the protective performance of ALD-derived ZrO_2_ barriers on Co–Cr alloys. Conformal barrier formation restricts electrolyte–substrate interaction and long-term metal-ion transport, while manufacturing-dependent substrate and interfacial characteristics modulate the final functional response. The cross symbols schematically indicate reduced metal-ion release through the ZrO_2_ barrier.

**Figure 6:**
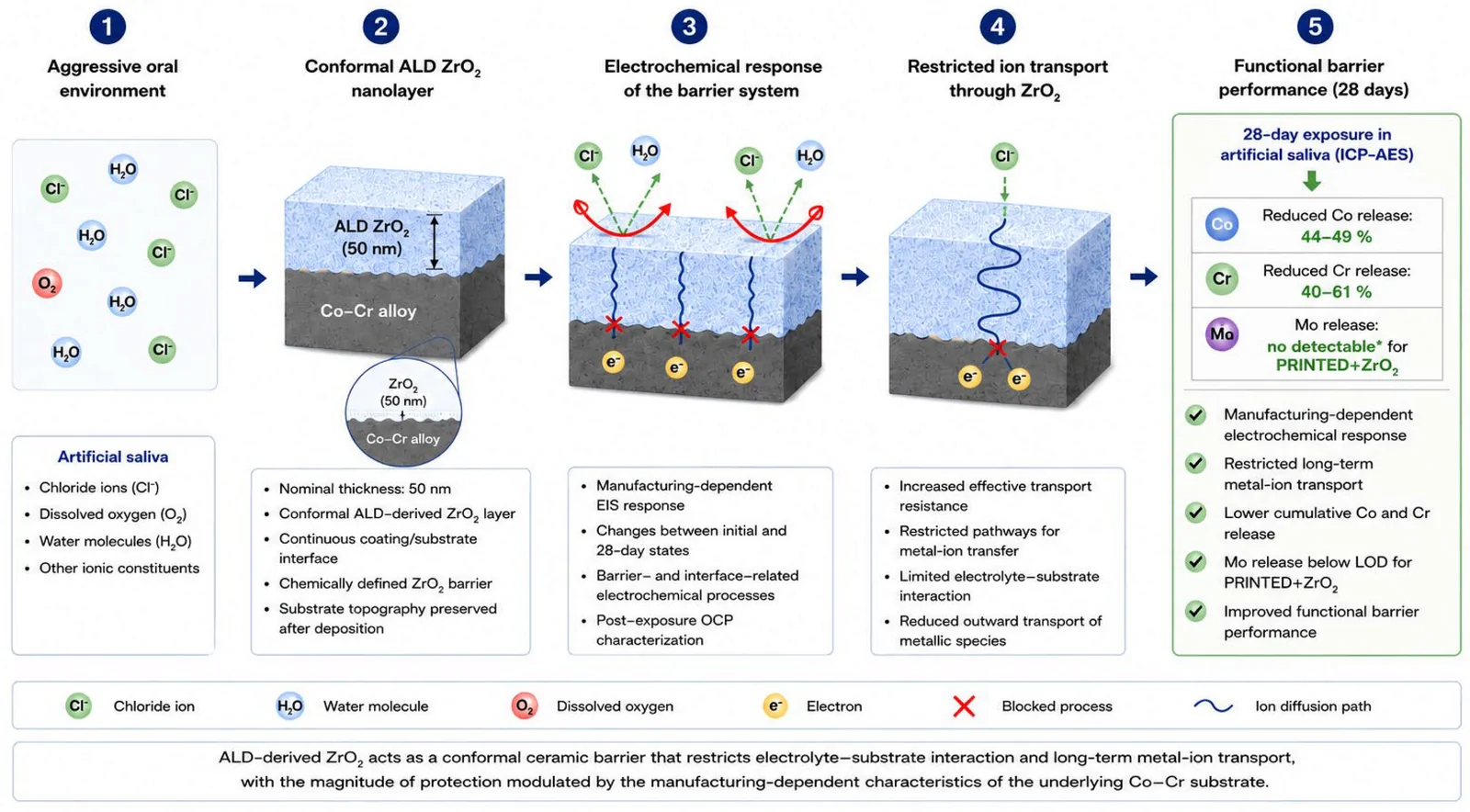
Integrated structure–barrier–release framework summarizing the experimental evidence obtained in the present study. Formation of a conformal ALD-derived ZrO_2_ barrier modifies the electrochemical response of the coating/substrate system and restricts long-term metal-ion transport during 28-day exposure to artificial saliva. The magnitude of the functional barrier effect depends on the manufacturing route of the underlying Co–Cr substrate, with PRINTED + ZrO_2_ exhibiting the lowest residual Co and Cr release and Mo release below the analytical detection limit. The curved red arrows and prohibition symbols schematically indicate restricted transport pathways and limited direct electrolyte access to the underlying Co–Cr substrate in the presence of the ZrO_2_ barrier.

Figure 5 and Figure 6 were generated using ChatGPT GPT-5.6 as conceptual schematic representations of the mechanisms and relationships discussed in the manuscript. The prompts instructed the model to create scientific schematics illustrating: (i) the proposed protective mechanism of an ALD-derived ZrO_2_ barrier on Co–Cr alloys, including restricted electrolyte–substrate interaction and reduced metal-ion release, with manufacturing-route-dependent differences among CAST, MILLED, and PRINTED substrates (Figure 5); and (ii) the structure–barrier–release framework linking structural and chemical characterization, electrochemical barrier response, and cumulative metal-ion release (Figure 6). The AI-assisted graphical outputs were subsequently reviewed and refined by the authors for scientific accuracy, annotation, and consistency with the experimental results. No experimental data or primary experimental images were generated by AI.

## 3. Results and Discussion

The experimental findings are presented following the sequence of evidence established in the experimental design. Rather than describing the results according to individual analytical techniques, the discussion is organised around consecutive stages of barrier-system verification, progressing from structural confirmation of barrier formation, through evaluation of electrochemical stability, to assessment of long-term functional performance. This approach enables the relationships between material structure, barrier integrity and ion-release suppression to be interpreted within a unified mechanistic framework.

### 3.1. Formation of the ALD-Derived Ceramic Barrier

The formation and physicochemical characteristics of the ALD-derived ZrO_2_ layer were evaluated using complementary chemical, structural, and microscopic techniques. All CAST, MILLED, and PRINTED specimens were subjected to the same ALD process, consisting of approximately 714 deposition cycles at 200 °C with a growth per cycle of approximately 0.7 Å cycle^−1^, corresponding to a nominal coating thickness of approximately 50 nm. Because systematic cross-sectional thickness mapping was not performed for all three substrate types, minor local substrate-dependent variations in the actual coating thickness cannot be excluded.

After Ar^+^ cleaning, residual carbon contents of 6.5, 8.0, and 10.2 at.% were determined for CAST + ZrO_2_, MILLED + ZrO_2_, and PRINTED + ZrO_2_, respectively. These route-dependent differences may reflect variations in the initial substrate surface state, adsorption of adventitious carbon, or residual carbon-containing species associated with the ALD process and subsequent sample handling. Although the highest residual carbon content was observed for the PRINTED + ZrO_2_ system, which also exhibited the lowest cumulative metal-ion release, the present XPS data do not establish a causal relationship between residual carbon content and barrier performance. Therefore, the carbon levels are interpreted as an additional indicator of route-dependent near-surface chemistry rather than as a direct determinant of the protective properties of the ZrO_2_ barrier.

**Figure 2:**
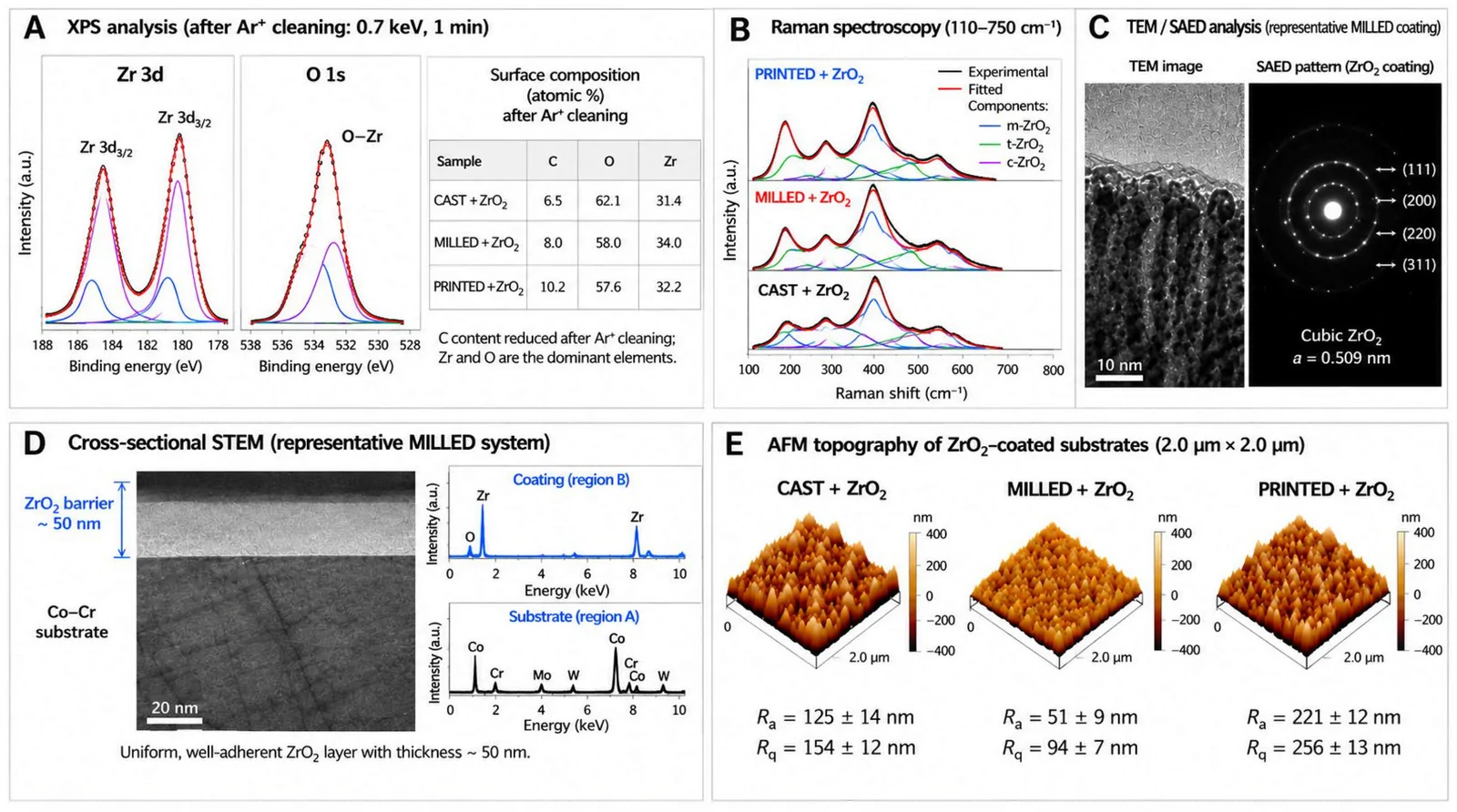
Integrated structural and chemical characterization of the ALD-derived ZrO_2_ barrier deposited on Co–Cr alloys manufactured by casting (CAST), milling (MILLED), and additive manufacturing (PRINTED). (**A**) XPS analysis confirming the chemical composition of the deposited ZrO_2_ layer after Ar^+^ cleaning. (**B**) Raman spectra with deconvolution into monoclinic (m), tetragonal (t), and cubic (c) ZrO_2_ components. (**C**) TEM/SAED analysis of the representative MILLED coating confirming its crystalline character. (**D**) Cross-sectional STEM analysis of the representative MILLED system illustrating the local morphology of the nanometre-scale ZrO_2_ layer and the coating/substrate interface. (**E**) AFM topography of the ZrO_2_-coated CAST, MILLED, and PRINTED substrates, showing manufacturing-route-dependent differences in surface morphology and the corresponding roughness parameters (Ra and Rq). Cross-sectional TEM/STEM observations of the PRINTED and MILLED systems provided complementary local evidence of the nanometre-scale ZrO_2_ layer and the coating/substrate interface, consistent with the application of the same nominal ALD protocol to all specimens. Equivalent initial cross-sectional verification was not available for the CAST system.

Raman spectroscopy demonstrated the multiphase crystalline character of the ALD-derived ZrO_2_ coatings (Figure 2B). Gaussian–Lorentzian analysis of the spectra in the 110–750 cm^−1^ range revealed contributions attributable to monoclinic (m), tetragonal (t), and cubic (c) ZrO_2_. The estimated monoclinic and tetragonal phase contributions were 6% and 66% for CAST + ZrO_2_, 12% and 65% for MILLED + ZrO_2_, and 4% and 72% for PRINTED + ZrO_2_, respectively. Thus, only modest manufacturing-route-dependent differences in the relative phase contributions were observed.

SAED provided complementary local crystallographic information. Diffraction acquired from a selected nanoscale region of the ZrO_2_ coating revealed a pattern consistent with cubic ZrO_2_, with a lattice parameter of a = 0.509 nm. Because SAED probes a spatially localized region, this observation confirms the local presence of a cubic ZrO_2_ domain rather than an exclusively cubic phase throughout the coating. The SAED result is therefore consistent with, rather than contradictory to, the larger-area Raman analysis, which demonstrates the multiphase character of the deposited ZrO_2_.

All CAST, MILLED, and PRINTED specimens were subjected to the same ALD protocol, consisting of approximately 714 cycles at 200 °C. With a growth per cycle of approximately 0.7 Å cycle^−1^, the process was designed to produce a nominal ZrO_2_ thickness of approximately 50 nm. Accordingly, the value of ~50 nm reported in this study should be understood as the nominal thickness defined by the ALD process parameters rather than as a precise thickness determined independently from individual cross-sectional TEM/STEM images.

AFM analysis revealed manufacturing-route-dependent differences in surface topography before and after ZrO_2_ deposition. For the CAST system, Ra and Rq increased from 67 ± 11 and 85 ± 9 nm for the uncoated substrate to 125 ± 14 and 154 ± 12 nm after ZrO_2_ deposition, respectively. For the MILLED system, the corresponding changes were smaller, from Ra = 44 ± 8 nm and Rq = 84 ± 10 nm to Ra = 51 ± 9 nm and Rq = 94 ± 7 nm. The most pronounced change was observed for the PRINTED system, for which Ra increased from 49 ± 8 to 221 ± 12 nm and Rq from 61 ± 4 to 256 ± 13 nm following ZrO_2_ deposition.

Taken together, the complementary analyses confirm ZrO_2_ formation on all three Co–Cr substrate types and demonstrate route-dependent differences in coating surface topography and near-surface chemistry. Cross-sectional TEM/STEM observations of the PRINTED and MILLED systems provided complementary local evidence of the nanometre-scale ZrO_2_ layer and the coating/substrate interface. Because equivalent initial cross-sectional verification was not available for CAST and systematic thickness mapping was not performed across the three substrate types, the nominal ~50 nm thickness is interpreted as a process-derived value rather than as an independently measured uniform thickness for each system.

### 3.2. Electrochemical Stability of the Barrier System

Following structural verification of the ALD-derived ZrO_2_ layer (Section 3.1), the electrochemical response of the coating/substrate systems was evaluated to determine how prolonged exposure to artificial saliva affected their barrier characteristics. Structural continuity alone cannot predict the electrochemical behaviour of an ultrathin ceramic coating; therefore, EIS responses obtained in the initial state and after 28 days of exposure were compared. Open-circuit potential (OCP) measurements performed after the exposure period provided complementary information on the electrochemical state of the investigated systems.

#### 3.2.1. Open-Circuit Potential After 28-Day Exposure

Open-circuit potential measurements performed after 28 days of exposure were used to characterize the electrochemical condition established at the barrier/electrolyte interface. The post-exposure OCP values differed among the investigated systems, indicating that the electrochemical state following prolonged contact with artificial saliva remained dependent on the manufacturing route and surface condition. Because OCP was determined after completion of the exposure period rather than monitored continuously, these measurements were interpreted as descriptors of the post-exposure electrochemical state and not of the temporal evolution of the interface.

#### 3.2.2. Electrochemical Impedance Response

**Figure 3:**
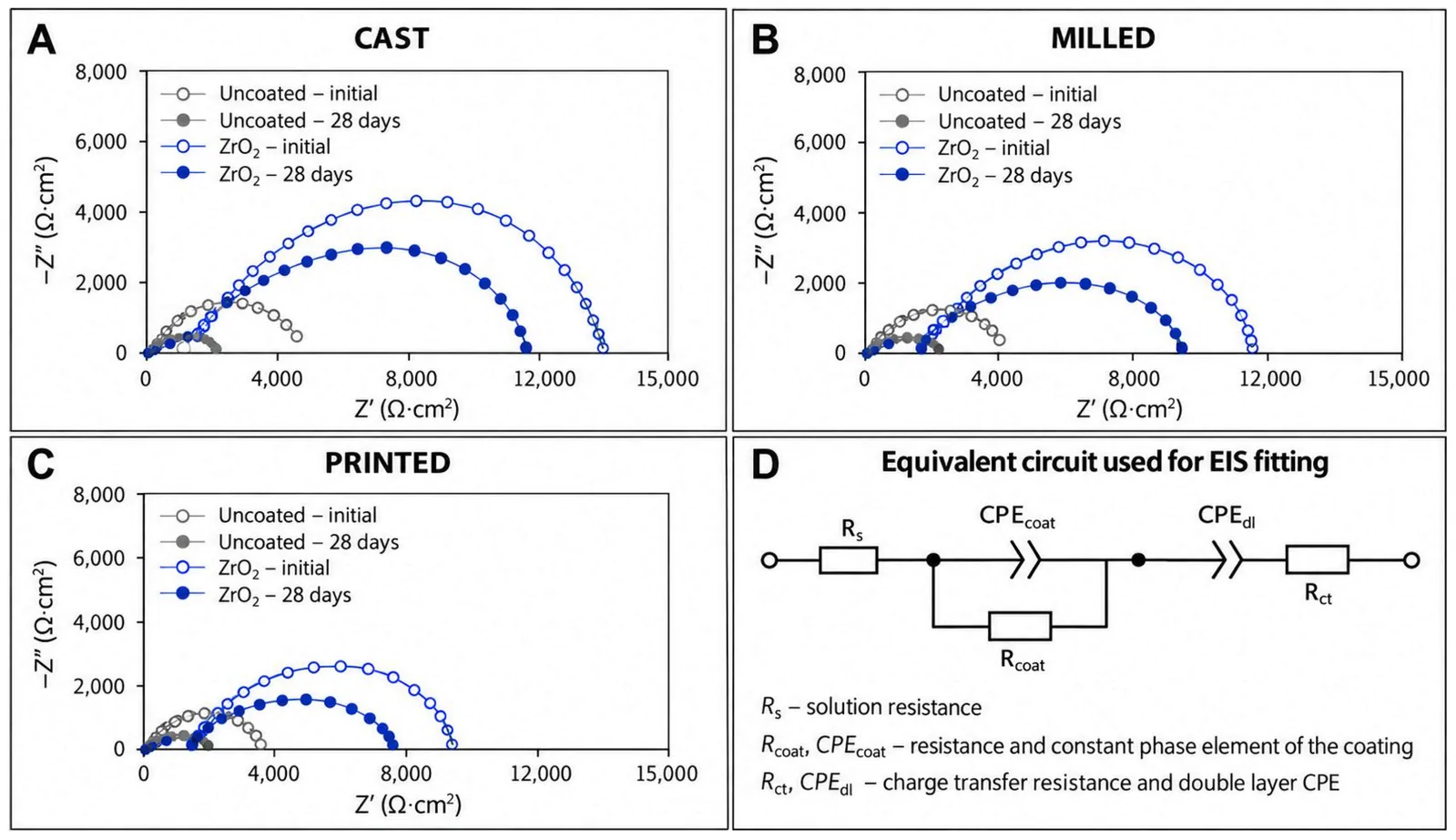
Electrochemical response of uncoated and ALD-derived ZrO_2_-coated Co–Cr systems before and after 28 days of exposure to artificial saliva. Representative Nyquist impedance spectra for (**A**) CAST, (**B**) MILLED, and (**C**) PRINTED systems. (**D**) Representative equivalent electrical circuit used for fitting the experimental EIS data.

A more detailed assessment of exposure-induced changes in electrochemical behaviour was obtained by comparing EIS spectra recorded in the initial state and after 28 days of immersion in artificial saliva (Figure 3). The Nyquist and Bode responses revealed manufacturing-dependent differences in both the magnitude and character of the impedance response. Thus, despite deposition of the same nominal 50 nm ZrO_2_ layer, CAST, MILLED and PRINTED substrates did not exhibit identical electrochemical behaviour, demonstrating that the response of the barrier cannot be considered independently of the underlying Co–Cr substrate.

Equivalent-circuit analysis provided further insight into the processes contributing to the observed impedance response. Depending on the investigated system and exposure condition, the fitted models included contributions associated with solution resistance (Rs), pore- or barrier-related resistance (Rpore), charge-transfer resistance (Rct), and constant-phase elements describing non-ideal capacitive behaviour. In systems exhibiting additional interfacial complexity, supplementary circuit elements were required to account for adsorption-related processes. The available post-exposure OCP values measured after the 28-day immersion period are summarized in Table 3. These values characterize the electrochemical state of the systems at the end of exposure and should not be interpreted as continuous OCP monitoring during the immersion period.

The use of different equivalent-circuit architectures for individual systems indicates that direct comparison of isolated fitted parameters should be approached with caution. Rather than treating Rct or Rpore as independent ranking parameters, the EIS response was interpreted mechanistically by considering the overall spectral characteristics, exposure-induced changes, and physical meaning of the circuit elements within each coating/substrate configuration.

For the CAST system, prolonged exposure produced a marked change in the impedance response. The uncoated alloy changed from an initial response containing porous-layer and interfacial contributions to a post-exposure response characterized by a different electrochemical architecture. Deposition of the ZrO_2_ layer further modified the impedance characteristics of the CAST system, demonstrating that the ceramic coating altered the electrochemical interface rather than simply adding a passive resistance component. The post-exposure behaviour nevertheless indicates that the response remained strongly dependent on the underlying CAST substrate and coating/substrate interface.

The MILLED systems exhibited a different electrochemical evolution. After 28 days, the uncoated MILLED alloy showed a pronounced contribution associated with the porous or surface layer, whereas the ZrO_2_-coated system retained a more complex interfacial response involving barrier- and charge-transfer-related processes. These observations demonstrate that the electrochemical effect of the deposited ZrO_2_ layer cannot be described solely by an increase or decrease in a single resistance parameter. Instead, ALD modifies the architecture of the electrochemical interface and the relative contributions of individual processes represented by the equivalent-circuit model.

The PRINTED system exhibited a distinct impedance response compared with CAST and MILLED, further demonstrating the influence of manufacturing route on the electrochemical behaviour of the complete barrier system. Despite application of an identical nominal ZrO_2_ layer, the additively manufactured substrate generated a different post-exposure response, indicating that substrate-dependent characteristics remained relevant after coating deposition.

#### 3.2.3. Influence of Manufacturing Route

The manufacturing-route dependence observed by EIS is consistent with the structural differences among the underlying Co–Cr substrates. Casting, subtractive machining and laser powder bed fusion generate different microstructural architectures and surface states, which may influence coating nucleation, coating/substrate interfacial characteristics and the accessibility of local transport pathways.

This interpretation is also consistent with the structural observations presented in Section 3.1. AFM analysis demonstrated that characteristic substrate-dependent topographical features remained evident following ALD. Because ALD growth is inherently conformal, the deposited layer follows the existing surface architecture rather than eliminating it. Substrate-dependent characteristics may therefore continue to influence the electrochemical response even after formation of the same nominally ~50 nm ZrO_2_ coating.

The EIS results further demonstrate why barrier performance cannot be ranked reliably on the basis of a single fitted resistance value. Different equivalent-circuit architectures represent different interfacial processes, and an apparently high Rct or Rpore value does not necessarily imply superior overall barrier performance when the underlying physical models differ. Mechanistic interpretation of the complete impedance response is therefore more appropriate for comparing the investigated systems.

#### 3.2.4. Electrochemical Implications for Barrier Performance

Taken together, comparison of the initial and 28-day EIS responses and the post-exposure OCP measurements demonstrates manufacturing-dependent electrochemical behaviour of the ALD-derived ZrO_2_/Co–Cr systems.

Importantly, electrochemical characterization represents an intermediate level of evidence within the proposed structure–barrier–release framework. Structural characterization establishes barrier formation, while EIS provides information on the electrochemical response of the barrier/substrate system before and after prolonged exposure. Neither approach, however, directly quantifies the amount of metallic species transferred from the substrate into the surrounding environment during the exposure period.

Consequently, electrochemical behaviour alone cannot provide the final functional verification of barrier effectiveness. The functional consequence of the observed barrier response was therefore assessed directly by quantitative determination of cumulative Co and Cr release for all three manufacturing routes and Mo release for the Mo-containing CAST and PRINTED alloys after 28 days of exposure to artificial saliva, as discussed in Section 3.3.

### 3.3. Functional Barrier Performance

**Figure 4:**
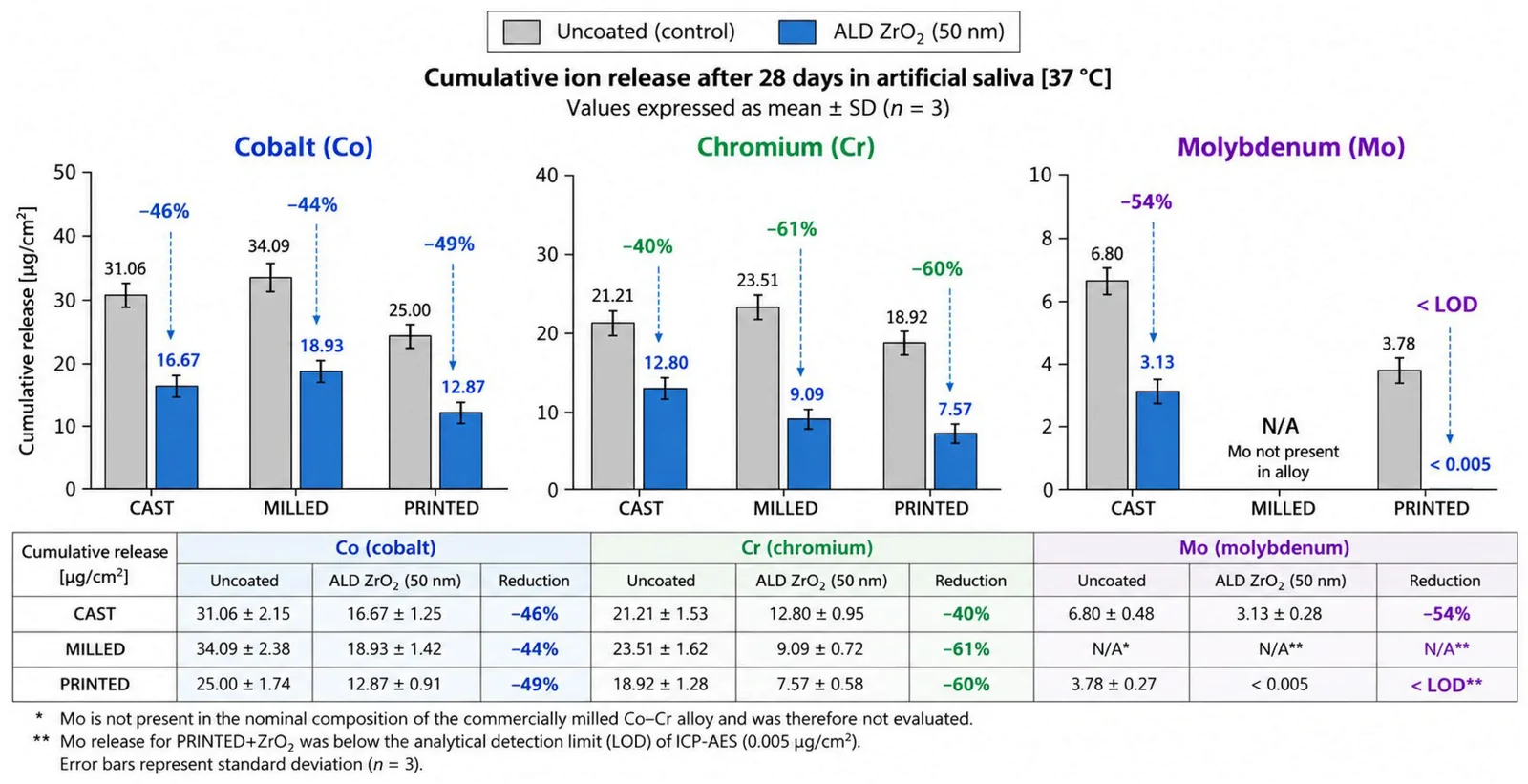
Cumulative Co, Cr, and Mo release from uncoated and nominally ~50 nm ALD ZrO_2_-coated Co–Cr alloys after 28 days of immersion in artificial saliva at 37 °C. Values are expressed as mean ± SD (*n* = 3) in µg/cm^2^. Percentage reductions following ZrO_2_ deposition are indicated for quantitatively determined values. Mo release was not evaluated for the MILLED system because Mo is not present in its nominal alloy composition; for PRINTED + ZrO_2_, Mo release was below the ICP-AES analytical detection limit (<0.005 µg/cm^2^).

Cumulative metal-ion release after 28 days was adopted as the principal functional descriptor of barrier performance because it quantifies metal transfer over the entire exposure period. Co and Cr release was evaluated for all three manufacturing routes, whereas Mo release was evaluated only for the Mo-containing CAST and PRINTED alloys. The cumulative release values and corresponding percentage reductions following ZrO_2_ deposition are summarized in Figure 4.

Figure 4 Cumulative Co, Cr, and Mo release from uncoated and nominally ~50 nm ALD ZrO_2_-coated Co–Cr alloys after 28 days of immersion in artificial saliva at 37 °C. Values are expressed as mean ± SD (*n* = 3) in µg/cm^2^. Percentage reductions following ZrO_2_ deposition are indicated for quantitatively determined values. N/A denotes “not applicable”; the asterisk annotations (*, **) are defined directly in the figure.

#### 3.3.1. Suppression of Cobalt Release

Deposition of the ALD-derived ZrO_2_ barrier reduced cumulative Co release for all three manufacturing routes (Figure 4). For CAST specimens, Co release decreased from 31.06 ± 2.15 µg/cm^2^ for the uncoated alloy to 16.67 ± 1.25 µg/cm^2^ after ZrO_2_ deposition, corresponding to a reduction of approximately 46%. For MILLED specimens, Co release decreased from 34.09 ± 2.38 to 18.93 ± 1.42 µg/cm^2^, representing an approximately 44% reduction. For PRINTED specimens, the corresponding decrease was from 25.00 ± 1.74 to 12.87 ± 0.91 µg/cm^2^, equivalent to an approximately 49% reduction.

Thus, ZrO_2_ deposition produced a consistent reduction in cumulative Co release across all investigated manufacturing routes, with relative reductions ranging from 44% to 49%. Among the coated systems, PRINTED + ZrO_2_ exhibited the lowest residual Co release, whereas MILLED + ZrO_2_ showed the highest. These results demonstrate a general barrier effect of the ALD-derived ZrO_2_ layer while simultaneously indicating that the final magnitude of Co release remained dependent on the underlying substrate.

#### 3.3.2. Suppression of Chromium and Molybdenum Release

The effect of ZrO_2_ deposition on Cr release was more strongly dependent on the manufacturing route (Figure 4). For CAST specimens, cumulative Cr release decreased from 21.21 ± 1.53 to 12.80 ± 0.95 µg/cm^2^, corresponding to an approximately 40% reduction. For MILLED specimens, Cr release decreased from 23.51 ± 1.62 to 9.09 ± 0.72 µg/cm^2^, representing an approximately 61% reduction. PRINTED specimens showed a comparable relative decrease, from 18.92 ± 1.28 to 7.57 ± 0.58 µg/cm^2^, corresponding to approximately 60%.

The relative reduction in Cr release therefore ranged from 40% to 61%, with the strongest suppression observed for the MILLED and PRINTED systems. In terms of absolute post-exposure values, however, PRINTED + ZrO_2_ again exhibited the lowest residual Cr release. This distinction indicates that relative reduction and final residual release represent complementary descriptors of barrier effectiveness.

Mo release provided additional information on the functional response of the investigated systems. For CAST specimens, cumulative Mo release decreased from 6.80 ± 0.48 to 3.13 ± 0.28 µg/cm^2^, corresponding to an approximately 54% reduction after ZrO_2_ deposition. For PRINTED specimens, Mo release decreased from 3.78 ± 0.27 µg/cm^2^ for the uncoated alloy to below the analytical detection limit (<LOD) following ZrO_2_ deposition. Mo release was not analyzed for the MILLED specimens, and no inference regarding the effect of the coating on Mo release can therefore be made for this manufacturing route.

The absence of detectable Mo release from PRINTED + ZrO_2_ provides additional evidence of the favourable functional response of this particular coating/substrate system under the applied experimental conditions.

#### 3.3.3. Influence of Manufacturing Route on Functional Barrier Performance

The ICP-AES results reveal two complementary aspects of barrier performance. First, deposition of the ZrO_2_ layer reduced cumulative Co and Cr release for all three manufacturing routes, demonstrating that the functional barrier effect was not restricted to a particular type of Co–Cr substrate. Second, both the magnitude of the reduction and the residual post-exposure ion release remained dependent on the manufacturing route.

This dependence is particularly evident when the absolute post-coating values are considered. Residual Co release decreased from 18.93 ± 1.42 µg/cm^2^ for MILLED + ZrO_2_ and 16.67 ± 1.25 µg/cm^2^ for CAST + ZrO_2_ to 12.87 ± 0.91 µg/cm^2^ for PRINTED + ZrO_2_. A similar pattern was observed for Cr, with residual values of 12.80 ± 0.95, 9.09 ± 0.72 and 7.57 ± 0.58 µg/cm^2^ for CAST + ZrO_2_, MILLED + ZrO_2_ and PRINTED + ZrO_2_, respectively. Together with Mo release below the detection limit, these results identify PRINTED + ZrO_2_ as the system exhibiting the lowest residual release of the analyzed metallic species.

This manufacturing-dependent response is consistent with the structural and electrochemical observations discussed in Section 3.1 and Section 3.2. Because ALD-derived coatings conform to the underlying substrate architecture, the final barrier response should not be regarded solely as an intrinsic property of the deposited ZrO_2_ layer. Instead, it reflects the behaviour of the complete coating/interface/substrate system, in which manufacturing-dependent substrate characteristics may influence interfacial behaviour and the effective pathways available for long-term ion transport.

#### 3.3.4. Functional Implications

These findings also clarify the complementary roles of electrochemical characterization and ICP-AES within the experimental strategy. EIS characterizes the electrochemical response of the coating/substrate system at defined measurement states, whereas cumulative ion release quantifies the net amount of metallic species transferred into the surrounding medium over the entire 28-day exposure period. Cumulative ion release therefore represents the principal functional endpoint of the present study.

Taken together, the results demonstrate that formation of the ALD-derived ZrO_2_ barrier translates into a measurable reduction in long-term metal-ion transport and that the magnitude of this effect depends on the manufacturing route of the underlying Co–Cr substrate. These observations provide the functional component of the proposed structure–barrier–release framework and establish the basis for the mechanistic interpretation developed in Section 3.4.

### 3.4. Mechanisms Governing Barrier Performance

The results presented in the preceding sections demonstrate a relationship between barrier formation, electrochemical response, and long-term suppression of metal-ion release. The protective performance of the ALD-derived ZrO_2_ coating should therefore be considered in terms of the interactions between the deposited layer, the underlying substrate, and the surrounding electrolyte.

Owing to the self-limiting surface reactions characteristic of ALD, this deposition technique enables highly conformal growth with precise control of nominal thickness, including on substrates with complex surface topographies [11,12,13]. In the present study, chemical, structural, and microscopic characterization confirmed the formation of the ZrO_2_ layer on all investigated Co–Cr substrates. AFM further showed that substrate-dependent topographical features remained evident after deposition, consistent with the conformal nature of ALD growth.

For nanometre-scale oxide coatings, corrosion-protective performance can be sensitive to local coating discontinuities and transport pathways that permit electrolyte access to the underlying metal [23,24]. In the present study, SEM observations were used to assess surface homogeneity and identify visible local defects or discontinuities, while cross-sectional TEM/STEM provided complementary local information on coating morphology and the coating/substrate interface. Within the examined regions, no large-scale delamination or macroscopic discontinuities were observed. However, these localized observations cannot exclude isolated nanoscale defects or pinholes over the entire coated surface. Accordingly, the route-dependent EIS response is interpreted as complementary functional evidence of differences in barrier integrity rather than as direct evidence of a particular defect population.

During immersion in artificial saliva, the ZrO_2_ surface interacts with water and dissolved ionic species. Interaction of zirconia with aqueous environments may involve dissociative adsorption of water and surface hydroxylation, depending on crystallographic surface and local surface chemistry [25]. The differences between the initial and 28-day EIS responses therefore reflect changes in the electrochemical state of the coated systems during exposure, without providing direct evidence of coating degradation or preservation at intermediate immersion times.

From a transport perspective, the deposited zirconia layer restricts ionic exchange between the electrolyte and the metallic substrate. Electrolyte species must traverse or circumvent the ceramic barrier to reach the alloy, while metallic species released at or near the substrate interface must cross the barrier before entering the surrounding medium. Localized corrosion of ALD-protected metallic substrates has been reported to develop preferentially at pre-existing coating defects [23], supporting the importance of limiting such transport pathways. Consistent with this mechanism, the cumulative ion-release results demonstrate that ZrO_2_ deposition restricted long-term metal transport during the 28-day exposure period.

The magnitude of this barrier effect nevertheless differed among the manufacturing routes. Although CAST, MILLED, and PRINTED substrates were subjected to the same nominal ALD process, differences remained in their electrochemical responses and residual Co and Cr release. Previous studies have similarly shown that the protective behaviour of ultrathin ALD oxide coatings can be influenced by substrate surface condition and coating–substrate interfacial characteristics [23,24,26]. Because ALD conformally follows the underlying surface architecture, manufacturing-dependent differences in substrate microstructure and topography may influence local coating formation and interfacial transport pathways.

EIS and ICP-AES provide complementary information in this context. EIS characterizes the electrochemical response of the coated systems at defined measurement states, whereas cumulative ion release quantifies the net amount of metallic species transferred into the surrounding medium throughout the exposure period. Their combined interpretation therefore links electrochemical barrier behaviour with long-term functional performance.

From a transport perspective, the ZrO_2_ layer acts as a physical barrier that limits direct electrolyte access to the metallic substrate and increases the effective transport resistance for species moving across the coating/substrate interface. The cumulative ion-release results indicate that this barrier effect was maintained sufficiently over the 28-day exposure period to reduce Co and Cr transfer for all three manufacturing routes and Mo transfer for the Mo-containing CAST and PRINTED alloys. The proposed mechanisms contributing to the protective behaviour of the ALD-derived ZrO_2_ barrier are schematically summarized in Figure 5.

Figure 5 Schematic representation of the proposed structure–barrier–release mechanism governing the protective performance of ALD-derived ZrO_2_ barriers on Co–Cr alloys. Conformal barrier formation restricts electrolyte–substrate interaction and long-term metal-ion transport, while manufacturing-dependent substrate and interfacial characteristics modulate the final functional response. Cross symbols schematically indicate reduced metal-ion release through the ZrO_2_ barrier, double upward arrows indicate comparatively higher Co and Cr release, and “<LOD” denotes Mo release below the analytical detection limit.

### 3.5. From Structure to Function

The complementary experimental methods used in this study provide three levels of information on barrier performance: structural and chemical characterization establishes ZrO_2_ formation, electrochemical measurements describe the response of the coated systems, and cumulative ion-release analysis quantifies long-term metal transfer. Their relationship is summarized in the structure–barrier–release framework shown in Figure 6.

Among 28 day exposure period. The combined results show that ALD-derived ZrO_2_ restricts metal-ion release from Co–Cr alloys, while the magnitude of this effect varies with the manufacturing route. This framework provides a materials-level basis for comparing protective coating/substrate systems prior to subsequent biological and risk-based evaluation.

## 4. Conclusions

This study establishes a structure–barrier–release framework for interpreting the long-term functional performance of low-temperature ALD-derived ZrO_2_ barriers deposited on Co–Cr alloys manufactured by casting, milling and additive manufacturing. By integrating structural, electrochemical and ion-release evidence, the proposed approach links barrier architecture and substrate-dependent electrochemical response with the functional suppression of long-term metal-ion transport under simulated oral conditions.

The principal conclusions of this study are as follows:

1. Low-temperature ALD enabled the formation of ZrO_2_ ceramic barriers on Co–Cr substrates produced by three fundamentally different manufacturing routes. All specimens were subjected to the same deposition protocol, corresponding to a nominal coating thickness of approximately 50 nm. Complementary chemical, structural, and microscopic characterization confirmed ZrO_2_ formation across the investigated systems, while cross-sectional TEM/STEM observations of the MILLED and PRINTED systems provided local information on the nanometre-scale coating morphology and coating/substrate interface. AFM observations further showed that characteristic substrate-dependent surface topography remained evident after deposition, consistent with the conformal nature of ALD growth. ZrO_2_ deposition reduced cumulative Co release by 44–49% and Cr release by 40–61% across all three manufacturing routes. Mo release was additionally reduced for the Mo-containing CAST and PRINTED alloys, reaching a level below the analytical detection limit (<0.005 µg cm^−2^) for PRINTED + ZrO_2_.
2. The electrochemical response of the ZrO_2_-coated systems remained dependent on the manufacturing route of the underlying Co–Cr substrate. Comparison of the initial and 28-day EIS responses, together with the available post-exposure OCP measurements, demonstrated manufacturing-dependent differences in the electrochemical behaviour of the investigated barrier/substrate systems.
3. ZrO_2_ deposition reduced cumulative Co release by 44–49% and Cr release by 40–61% across all three manufacturing routes. Mo release was additionally reduced for the Mo-containing CAST and PRINTED alloys, reaching a level below the analytical detection limit (<0.005 µg cm^−2^) for PRINTED + ZrO_2_.
4. The proposed structure–barrier–release framework integrates complementary levels of evidence into a unified strategy for evaluating functional ceramic barriers. Structural and chemical characterization establishes barrier formation, electrochemical measurements characterize the response of the barrier/substrate system, and cumulative ion-release analysis quantifies the resulting long-term functional outcome. Their combined interpretation provides a more comprehensive assessment of barrier performance than any of these approaches considered independently.

## Figures and Tables

**Table 1:** Literature gap analysis and scientific contribution of the present study.

Reference	Main Focus	Limitation	Contribution of the Present Study
[1,5]	Biological significance of metal ion release from dental and biomedical alloys	No surface-engineering strategy proposed to reduce long-term Co/Cr release	Demonstrates substantial suppression of cumulative Co and Cr release using ALD ZrO_2_ coatings
[7,8,9]	Corrosion behavior of Co–Cr alloys in artificial saliva	Primarily electrochemical evaluation; ion release treated as secondary outcome	Uses 28-day cumulative ion release as a primary functional descriptor of barrier performance
[2]	Co–Cr alloys in prosthodontics	No analysis of advanced nanometric barrier coatings	Evaluates conformal ALD ZrO_2_ films on prosthetic Co–Cr alloys
[11,12,13]	Fundamentals of atomic layer deposition	Not focused on dental Co–Cr substrates or saliva-like environments	Applies low-temperature ALD technology to clinically relevant Co–Cr prosthetic materials
[17,18]	Zirconia as a dental biomaterial	Focus on bulk zirconia rather than nanometric ALD barrier films	Investigates ultrathin ALD-deposited ZrO_2_ as a protective interfacial layer
[15,16,19]	Improvement of corrosion resistance	Limited correlation between coating structure and long-term ion release	Establishes a structure–barrier–release relationship
[15]	Surface properties and bacterial adhesion of ALD ZrO_2_-coated Co–Cr alloys	Did not investigate long-term metal ion release	Extends evaluation toward long-term Co/Cr release suppression
[16]	ALD ZrO_2_ coatings on Co–Cr alloys in the context of biological safety and MDR-oriented design	Long-term ion release was not used as the principal performance metric	Demonstrates ion-release reduction as a functional measure of coating effectiveness
[20]	Multifactorial etiology of denture stomatitis	Limited focus on material-related ion release factors	Addresses reduction of Co/Cr transfer as a strategy to minimize one material-dependent risk factor
[21,22]	Biological safety and risk assessment of medical devices	Do not provide engineering solutions for reducing ion release	Presents a surface-engineering approach supporting improved biological safety profiles

**Table 2:** Nominal chemical composition of the investigated Co–Cr dental alloys according to the manufacturers (wt.%).

	GialloyPA Type 5	CopraBond K Type 4	EOS CoCr RPD Type 5
Co	62	61	63.8
Cr	30	28	24.7
Mo	5.5	-	5.1
Si	1.0	1.65	1.0
Mn	0.6	0.3	max 0.1
W	-	8.5	5.4
C	0.6	0.1	-
Fe	-	0.3	max 0.5

**Table 3:** Post-exposure open-circuit potential (OCP) values measured after 28 days of immersion in artificial saliva.

Manufacturing Route	Surface Condition	EIS States Compared	Post-Exposure OCP (mV vs. Ag/AgCl)	Main Electrochemical Observation
CAST	Uncoated	Initial/28 d	−173 ± 10 mV vs. Ag/AgCl	Exposure-induced modification of the impedance response
CAST	ZrO_2_-coated	Initial/28 d	151 ± 8 mV vs. Ag/AgCl	Barrier-related response modified after prolonged exposure
MILLED	Uncoated	Initial/28 d	−224 ± 8 mV vs. Ag/AgCl	Pronounced exposure-dependent change in impedance characteristics
MILLED	ZrO_2_-coated	Initial/28 d	−137 ± 6 mV vs. Ag/AgCl	Complex interfacial response requiring an adsorption-related contribution
PRINTED	Uncoated	Initial/28 d	Numerical value not available	Manufacturing-specific impedance response before and after exposure
PRINTED	ZrO_2_-coated	Initial/28 d	Numerical value not available	Distinct coating/substrate response following prolonged exposure

Abbreviations: EIS, electrochemical impedance spectroscopy; OCP, open-circuit potential.

## Data Availability

The original contributions presented in this study are included in the article. Further inquiries can be directed to the corresponding author.

## Abbreviations

The following abbreviations are used in this manuscript:

**Abbreviation**	**Definition**
AFM	Atomic Force Microscopy
ALD	Atomic Layer Deposition
CAST	Conventionally Cast Co–Cr Alloy
Co–Cr	Cobalt–Chromium
DMLS	Direct Metal Laser Sintering
EIS	Electrochemical Impedance Spectroscopy
ICP-AES	Inductively Coupled Plasma Atomic Emission Spectroscopy
LOD	Limit of Detection
LPBF	Laser Powder Bed Fusion
MDR	Medical Device Regulation
MILLED	CAD/CAM-Milled Co–Cr Alloy
OCP	Open-Circuit Potential
PRINTED	Additively Manufactured Co–Cr Alloy
SAED	Selected-Area Electron Diffraction
SEM	Scanning Electron Microscopy
STEM	Scanning Transmission Electron Microscopy
TEM	Transmission Electron Microscopy
XPS	X-ray Photoelectron Spectroscopy
XRD	X-ray Diffraction
ZrO_2_	Zirconium Dioxide
